# Predicting Response to [177Lu]Lu-PSMA Therapy in mCRPC Using Machine Learning

**DOI:** 10.3390/jpm14111068

**Published:** 2024-10-23

**Authors:** Kaiyuan Gong, Baptiste Magnier, Salomé L’hostis, Fanny Borrely, Sébastien Le Bon, Nadine Houede, Adel Mamou, Laurent Maimoun, Pierre Olivier Kotzki, Vincent Boudousq

**Affiliations:** 1 Department of Computer Science & Artificial Intelligence, IMT Mines Ales, 30100 Ales, France; kaiyuan.gong@etu.mines-ales.fr; 2 Service de Médecine Nucléaire, Centre Hospitalier Universitaire de Nîmes, Université de Montpellier, 34295 Nîmes, France; 3 EuroMov Digital Health in Motion, Université de Montpellier, IMT Mines Ales, 30100 Ales, France; 4 Faculty of Medicine, Université de Montpellier, Nimes Caremeau Campus, 34295 Nîmes, France; 5 Institut de Recherche en Cancérologie de Montpellier (IRCM), INSERM U1194, Université de Montpellier, Institut Régional du Cancer de Montpellier (ICM), 34298 Montpellier, France; 6 Service d’Oncologie Centre Hospitalier Universitaire de Nîmes, Université de Montpellier, 34295 Nîmes, France; 7 Service de Médecine Nucléaire, Hôpital Lapeyronie, CHU Montpellier, 34295 Montpellier, France

**Keywords:** prostate cancer (PC), prostate specific membrane antigen (PSMA), FDG (fluorodeoxyglucose), Choline, Lu-177, radioligand therapy (RLT), machine learning (ML), response prediction

## Abstract

Background/Objectives: Radioligandtherapy (RLT) with [177Lu]Lu-PSMA has been newly introduced as a routine treatment for metastatic castration-resistant prostate cancer (mCRPC). However, not all patients can tolerate the entire therapeutic sequence, and in some cases, the treatment may prove ineffective. In real-world conditions, the aim is to distinguish between patients who fully benefit from treatment (those who respond effectively and tolerate the entire therapeutic sequence) and those who do not respond or cannot tolerate the entire sequence. This study explores predictive factors to distinguish between fully beneficial RLT treatment patients (FBTP) and not fully beneficial RLT treatment patients (NFBTP). The objective was to enhance the understanding of predictive factors influencing RLT effectiveness and to highlight the significance of machine learning in optimizing patient selection for treatment planning. Methods: Data from 25 mCRPC patients, categorized as FBTP (11) or NFBTP (14) to RLT, were analyzed. The dataset included clinical, imaging, and biological parameters. Data analysis techniques, including exploratory data analysis and feature engineering, were used to develop machine learning models for predicting patient outcomes. Results: Imaging data analysis revealed statistically significant differences in the renal uptake intensity of Choline between the two groups. A discordance of FDG+ and PSMA− was identified as a potential indicator of NFBTP. The integration of biological data enhanced the model’s predictive capability, achieving an accuracy of 0.92, a sensitivity of 0.96, and a precision of 0.96. Adding blood parameters like neutrophils, leukocytes, and alkaline phosphatase greatly increased prediction accuracy. Conclusions: This study emphasizes the significance of an integrated approach that merges imaging and biological data, thereby augmenting the predictive accuracy of patient outcomes in RLT with [177Lu]Lu-PSMA. In particular, including Choline PET among the imaging parameters provides unique insights into the predictive factors affecting RLT efficacy. This approach not only deepens the understanding of predictive factors but also underscores the utility of machine learning in refining the patient selection process for optimized treatment planning.

## 1. Introduction

Prostate Cancer (PC) ranks as the second most commonly diagnosed cancer in males and is the fifth leading cause of cancer-related mortality [1]. Approximately 10 to 30% of PC cases progress to castration-resistant prostate cancer (CRPC), with many evolving further into metastatic CRPC (mCRPC). Prostate-specific membrane antigen (PSMA), a transmembrane glycoprotein, is frequently overexpressed in prostate cancer cells and is associated with early recurrence, resistance to castration therapy, and poor prognosis [2,3]. Metastatic lesions in patients with mCRPC typically exhibit PSMA positivity [4]. Radioligand therapies (RLTs) target PSMA with the β-emitting [177Lu]Lu-PSMA-617, delivering radiation directly to cancer cells and their surroundings. This leads to better overall survival rates than standard treatments [5], and outperforms cabazitaxel chemotherapy in biochemical response [6].

[177Lu]Lu-PSMA-617, which received approval from the U.S. Food and Drug Administration and the European Medicine Agency in 2022 for treating progressive PSMA-positive metastatic CRPC, has shown efficacy, although not all PSMA-positive metastatic patients respond to this PSMA-targeted radioligand therapy (PSMA-RLT) [7].

Studies involving [177Lu]Lu-PSMA-617 and [177Lu]Lu-PSMA-I&T have reported that only 32 to 60% of patients experienced a decrease in prostate-specific antigen (PSA) levels by 50% or more [8]. Moreover, while previous research has identified pre-therapeutic clinical, laboratory, or imaging markers—such as [68Ga]Ga-PSMA-11 PET/CT (Positron Emission Tomography/Computed Tomography) findings—to predict the response to [177Lu]Lu-PSMA-617 treatment [9], there has been a limited exploration of alternative PET imaging findings [10]. The PSMA-RLT sequence consists of six [177Lu]Lu-PSMA-617 injections spaced 6 weeks apart, but not all patients can tolerate the full course of treatment. Two groups of patients are defined: those who were able to receive all six injections and for whom the treatment was effective, i.e., the fully beneficial RLT treatement patients (FBTP), and those for whom the treatment was not effective or who were unable to complete the entire therapeutic, named as not fully beneficial RLT treatment patients (NFBTP). This study seeks to identify predictive factors of the treatment response to [177Lu]Lu-PSMA-617 using [18F]Choline and [68Ga]Ga-PSMA-11 PET data.

Predicting patient response to RLT is crucial for optimizing treatment efficacy and identifying individuals who will benefit the most. Leveraging machine learning techniques to analyze extensive datasets of clinical and biomarker information offers promising avenues for enhancing prediction accuracy. Previous studies have demonstrated the effectiveness of machine learning models in forecasting responses to RLT based on early treatment data, as in [11]. Given the classification nature of the task and the limited size of the dataset, a stacking model combining Bayesian classifiers and Support Vector Classifiers (SVC) was opted for due to their enhanced performance, making them well suited for handling overfitting and small datasets [12]. To refine the proposed approach, thorough feature engineering was conducted, employing various algorithms to rank features and selecting the most relevant ones through a consensus approach [13,14]. The model underwent rigorous training using Leave-One-Out Cross-Validation (LOOCV) to ensure the reliability and accuracy of the predictions.

In addressing these challenges, a solution for predicting treatment responses in mCRPC patients was developed, along with a systematic framework. This framework demonstrates the entire process from unstructured data collection to feature selection and classification/prediction tasks using machine learning models. It can be broadly applied to similar small-sample, multi-feature classification/prediction tasks. The detailed process of this framework is described in the Materials and Methods section.

## 2. Materials and Methods

This section outlines the strategies and methods in evaluating the effect of PSMA-RLT for patients with mCRPC. The procedure of selecting patients and imaging and treatment protocol, as well as the evaluation of treatment effects and treatment outcomes, are described. Additionally, this section details the comprehensive data analysis and model development strategies employed to enhance the predictive accuracy of treatment responses and introduces a framework that guides the entire process.

### 2.1. Experimental Protocol

#### 2.1.1. Patient Cohorts

The data of mCRPC patients who were referred to the Department of Nuclear Medicine of the Nîmes University Hospital, France, were retrospectively analyzed between January 2022 and May 2023 for [177Lu]Lu-PSMA-617. PSMA-RLT therapy was recommended for all mCRPC patients after a discussion by an interdisciplinary tumor board after they failed to respond to the other available standard therapies. Prior to the [177Lu]Lu-PSMA-617 therapy, patients underwent [18F]Choline and [68Ga]Ga-PSMA-11 PET scans. Additionally, a subset of 9 patients also underwent a [18F]FDG PET scan. Only 25 patients (median age 72.1 years) with PSMA-positive metastases were treated by PSMA-RLT and included in the study.

Table 1 summarizes the principal characteristics of the patients included in the study.

#### 2.1.2. PET Imaging Protocols

The PET scan acquisitions were conducted using a Discovery PET/CT 710, GE Medical Systems (3000 N. Grandview Blvd. Waukesha, WI 53188, USA). and were performed in accordance with the EANM procedure guidelines for radiopharmaceuticals [15,16]. Imaging was conducted 60 min after the injection of 3 MBq/kg of [18F]FDG, 2.4 MBq/kg of [18F]Choline, and 2 MBq/kg of [68Ga]Ga-PSMA-11 at specified doses, respectively. If there were no contraindications, 20 mg of furosemide was intravenously injected shortly before the administration of the radiopharmaceutical. The imaging strategy included a CT topogram, followed by a low-dose attenuation-correction CT scan and PET acquisition. This was then followed by an additional intravenous contrast-enhanced diagnostic CT and a deep inspiration chest CT. In cases of contraindication to the contrast medium, a diagnostic CT scan was performed in place of the attenuation-correction CT scan with an additional deep inspiration chest CT. A lesion was defined as positive if confirmed by two experienced physicians. Regions of Interest (ROI) were applied for bone, sub or supradiaphragmatic lymph nodes, pelvic tissue invasion, liver, lung, or other tissues, where SULpeak, SULmax, SULmin, SULmean, and standard deviation were measured. For cases with multiple organ lesions, measurements were recorded for the most and least intense lesions. Due to the potential for extensive bone invasion, automatic ROI volume measurements were found to be unreliable. The extent of bone invasion was visually assessed. The skeleton was divided into ten parts: skull, cervical, thoracic and lumbar rachis, ribcage, scapula, pelvis, femur, and humerus. Each segment was evaluated using a score ranging from 0 to 10, and the overall bone extension was scored from 0 to 100. Background measurements were equally applied to the liver, kidneys, and salivary glands.

#### 2.1.3. [177Lu]Lu-PSMA-617 Protocol

According to the EANM/SNMMI procedure guidelines [17], PSMA-RLT typically comprises six cycles of intravenous administration of 7.4 GBq of [177Lu]Lu-PSMA-617 (AAA, Novartis) at six-week intervals. Hydration was ensured by an intravenous infusion of 1000 mL of 0.9% saline at a rate of 250 mL/h, starting 60 min before and continuing for a few hours after administration. A clinical and biological evaluation (complete blood counts, liver and kidney function tests, PSA levels) was performed one week before and three weeks after RLT. Additionally, a second [68Ga]Ga-PSMA-11 whole-body PET scan was conducted for all patients to visually evaluate the therapy response four to six weeks after the third PSMA-RLT cycle. Not all patients were able to complete six cycles of treatment due to deteriorating health.

### 2.2. PSMA-RLT Response Evaluation

After the therapeutic sequence and based on previous studies [18,19], the criteria for assessing the response incorporate clinical and biological considerations. As a biological criterion for a favorable response, a reduction in the serum PSA levels of more than 50% three weeks after the last [177Lu] Lu-PSMA-617 injection is considered. FBTP are defined as patients who tolerated all 6 treatment cycles with a final reduction in PSA of at least 50%. NBTP are defined as patients with a final PSA reduction of less than 50% or who were unable to tolerate the entire therapeutic sequence.

### 2.3. Data Analysis and Model Development

**Figure 1:**
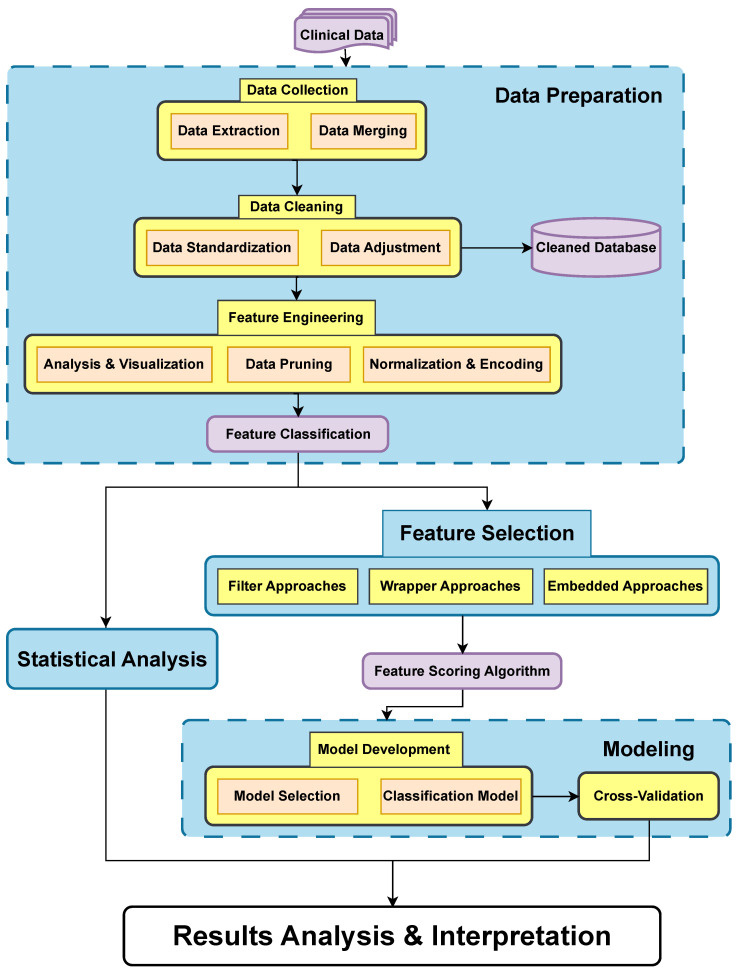
Data analysis and model development workflow diagram.

This section rigorously engages in comprehensive data analysis and model development to predict treatment responses in metastatic castration-resistant prostate cancer using advanced machine learning techniques. From meticulous data preparation and detailed statistical analyses to sophisticated feature selection and model optimization, each phase has been methodically structured to enhance the predictive capabilities of the proposed models. The integration of these methodologies ensures a robust and reliable framework that not only optimizes feature utilization but also maximizes model performance across various machine learning algorithms, paving the way for significant improvements in personalized cancer treatment strategies. The systematic framework proposed can be broadly applied to similar small-sample, multi-feature classification/prediction tasks. The detailed process of this framework is described below and visually represented in Figure 1.

#### 2.3.1. Data Preparation

##### Data Collection and Integration

All data types included in the study broadly consist of radiomics features, clinical parameters, and biological parameters. Radiomics features encompass imaging parameters, capturing both the first and higher order statistical features (mean, minimum, maximum, peak values, etc.) of regions of interest. Scores from visually assessed bone invasion across ten skeletal segments were recorded, while invasions of pelvic tissues, liver, and lungs were documented as binary yes/no variables. Lymph node involvement was quantified numerically. Additionally, the consistency and fixed states of the PSMA, Choline, and FDG tracers were documented. Clinical parameters included patient age, Gleason score, TNM staging, and WHO classification. Biological parameters involved comprehensive evaluations such as PSA levels, complete blood counts, and liver and kidney function tests.

The data were meticulously extracted, organized, and integrated from frontline medical records to form an initial dataset, complete with detailed data documentation. Based on the PSMA-RLT response evaluation, out of the 25 patients, 11 and 14 patients have been classified as Fully Beneficial Treatment Patients (FBTP) and 14 as Not Fully Beneficial Treatment Patients (NFBTP). A summary of these parameters is shown in Table 2. The parameters were categorized into continuous and categorical variables. These variables were then combined into feature vectors for machine learning analysis.

##### Data Cleaning

In the data cleaning phase of the study, several essential steps were undertaken to ensure the quality and utility of the data for machine learning applications.

First, data representations were standardized based on expert consensus, unifying variables to address issues of data sparsity and inconsistencies in recording. Binary variables were converted into Boolean or 0/1 formats. For textual data, a predefined coding scheme was utilized to extract useful information; for example, information on tracer differences such as PSMA and FDG was encoded numerically: −1 for missing data, 0 for no difference, 1 for PSMA−, FDG+ presence, and 2 for PSMA+, FDG− presence. This transformation streamlined the computational modeling process and enabled clearer statistical analysis.

Additionally, routine checks for missing data were conducted, with appropriate strategies for imputation or exclusion applied based on the data’s nature and the intended analysis, ensuring the dataset’s integrity and reliability was maintained. These data cleaning efforts were pivotal in preparing a refined dataset ready for further analysis, supporting the overall goal of leveraging frontline medical data in machine learning frameworks to improve treatment predictions and outcomes.

##### Feature Engineering

In the feature engineering phase, critical steps were undertaken to optimize the dataset for various machine learning algorithms.

First, irrelevant or redundant variables were removed, and potentially useful ones were explored and added based on their characteristics. For example, variables such as dates and post-treatment follow-up were removed as they could not serve as predictive factors.

To facilitate the use of these variables in algorithmic modeling, continuous variables were standardized using the Z-score method, resulting in a mean of 0 and a standard deviation of 1. This made different variables comparable on the same scale. Categorical variables underwent ordinal encoding, based on their order, and one-hot encoding, creating binary columns for each category. This standardization and encoding not only enhanced the predictive power of the models but also ensured consistency and comparability across different analytical approaches.

#### 2.3.2. Statistical Analysis

In this section, the focus was on hypothesis testing and statistical inference. These analyses were crucial for identifying potential variables and features, guiding feature selection, and enhancing model interpretability and confidence.

##### Mann–Whitney U Test

To compare the two classes with respect to continuous variables, the Mann–Whitney U test was employed (a non-parametric test). For each continuous variable, data were split along the value of class label (for two classes, it was FBTP/NFBTP), and the distributions of two groups were compared. The corresponding hypotheses are as follows:

- $H_{0}$: The distributions of the two groups are equal.
- $H_{a}$: The distributions of the two groups are not equal.

Concerning the calculation of the Mann–Whitney U statistic, it is determined as follows:

(1) $$U=n_{1}n_{2}+\frac{n_{1}\left(n_{1}+1\right)}{2}-R_{1},$$

where

- $n_{1}$ and $n_{2}$ represent the sample sizes of the two groups.
- $R_{1}$ is the sum of the ranks for the first group (class NFBTP).

An appropriate *p*-value is then calculated from the U statistic using statistical tables. If this *p*-value is low (*p* < 0.05), it indicates a rejection of the null hypothesis, suggesting that the two groups have different distributions of scores.

##### Chi-Square Test

For categorical variables, the Chi-Square ($\chi^{2}$) test was employed to assess the independence between each categorical variable and the binary class label (FBTP/NFBTP). The hypotheses for the test are as follows:

- $H_{0}$: The two categorical variables are independent.
- $H_{a}$: The two categorical variables are not independent.

The $\chi^{2}$ statistic is calculated by

(2) $$\chi^{2}=\sum \frac{{\left(O_{i}-E_{i}\right)}^{2}}{E_{i}},$$

where

- $O_{i}$ represents the observed frequency for category *i*.
- $E_{i}$ is the expected frequency for category *i*, calculated under the assumption that the two variables are independent.

Thereafter, $\chi^{2}$ is used to obtain the *p*-value from the statistical tables. A small *p*-value (<0.05) leads to a rejection of the null hypothesis of independence in favor of the alternative hypothesis that the two categorical variables are related.

#### 2.3.3. Feature Selection

Feature selection was crucial in pinpointing the most relevant features for the classification tasks, enhancing both model efficiency and accuracy. The process utilized established techniques divided into filter, wrapper, and embedded approaches.

In each approach, various methods are employed to score and rank the importance of the features. A comprehensive scoring algorithm integrates multiple feature evaluation methods to assess their significance. The ranks obtained by each feature in different feature selection algorithms, including correlation coefficients in Correlation-Based Methods, mutual information values in Mutual Information-Based Methods, and feature importance metrics in the Random Forest method, reflect their significance.

The algorithm draws inspiration from the Borda count method, transforming the rankings of the top 50 continuous variables and all categorical variables into scores, with higher scores assigned to higher-ranked features. The total scores for each feature are then calculated by summing up its scores across different algorithms, and the features are ranked based on their total scores, where a higher score denotes greater importance (see Algorithm 1).
**Algorithm 1** Comprehensive Feature Scoring Algorithm Initialize FeatureScores as empty dictionary RankingLists← Retrieve feature ranking lists **for all** ranking in RankingLists **do**  **for all** (feature,rank) in ranking **do**   N← total features in ranking   score←N−rank+1   **if** feature not in FeatureScores **then**    FeatureScores[feature]←0   **end if**   FeatureScores[feature]←FeatureScores[feature]+score  **end for** **end for** SortedFeatures←Sort FeatureScores by values descending **return** SortedFeatures

##### Filter Approaches

Filter methods assess the importance of features using statistical measures, independent of any machine learning models. In this context, applied techniques include the following:

**Correlation-Based Methods:** The proposed methodology combines three different correlation-based methods to assess the importance of features. Each method produces an individual importance score for each feature. Finally, the scores are aggregated to obtain the final ranks of the features.

**Spearman Rank Correlation:** A non-parametric measure of rank correlation that assesses how well the relationship between two variables can be described using a monotonic function:

(3) $$\rho =1-\frac{6\sum d_{i}^{2}}{n(n^{2}-1)},$$

where $d_{i}$ is the difference between the ranks of each observation and *n* is the total number of observations.

**Point-Biserial Correlation:** this measures the relationship between a continuous variable and a binary variable:

(4) $$r_{pb}=\frac{M_{1}-M_{2}}{\sigma }\sqrt{\frac{n_{1}n_{2}}{n(n-1)}},$$

where $M_{1}$ and $M_{2}$ are the means of the two groups, σ is the standard deviation of the total sample, $n_{1}$ and $n_{2}$ are the sample sizes of the two groups, and *n* is the total sample size.

**Binary Categorical Regression Assessing Correlation (BCRAC):** This measures the relative association of binary categorical variables compared to other features and is computed using logistic regression to assess predictive power. The importance of a feature is determined by its coefficient in the logistic regression model. The logistic regression model is defined as follows:

(5) $$\mathrm{logit}\left(P\left(Y=1|X\right)\right)=\beta_{0}+\beta_{1}x_{1}+\beta_{2}x_{2}+\cdots +\beta_{p}x_{p},$$

where P(Y=1|X) is the probability of the binary outcome *Y* given the predictors $X=(x_{1},x_{2},\ldots ,x_{p})$, $\beta_{0}$ is the intercept term, and $\beta_{1},\beta_{2},\ldots ,\beta_{p}$ are the coefficients for the predictors. The significance of each feature is assessed based on the magnitude and statistical significance of its corresponding coefficient $\beta_{i}$.

The overall importance score for a feature $x_{i}$ can be derived from the absolute value of its coefficient:

(6) $$\mathrm{Importance}\left(x_{i}\right)=\left|\beta_{i}\right|.$$

Features with higher absolute coefficients are considered more important as they have a greater impact on the predicted outcome.

**Mutual Information-Based Methods:** This category included methods like Information Gain and Minimum Redundancy Maximum Relevance (MRMR), which evaluate information sharing between features to reduce redundancy.

**Information Gain (IG):** A univariate method that selects branches of a decision tree that contribute the most towards prediction. The features that contain the most information are selected from the dataset. This is calculated as follows:

(7) IG(T,a)=H(T)−H(T|a),

where IG(T,a) is the information gain of feature *a* with respect to the target *T*, H(T) is the entropy of *T*, and H(T|a) represents the conditional entropy of the target given feature *a*.

**Minimum Redundancy Maximum Relevance (MRMR):** Selects features that that are maximally relevant to the target and minimally redundant (dependent) with each other. It is calculated as follows:

(8) $$\mathrm{MRMR}=\max\left[I\left(X;Y\right)-\frac{1}{\left|S\right|}\sum_{x_{i}\in S}I\left(x_{i};X\right)\right],$$

where I(X;Y) is the mutual information between the feature *X* and the target *Y*, and $\frac{1}{\left|S\right|}\sum_{x_{i}\in S}I\left(x_{i};X\right)$ is the average mutual information between the feature *X* and the already selected features in subset *S*.

##### Wrapper Approaches

Wrapper methods iteratively refine feature subsets using predictive models, evaluating and adjusting features based on their contribution to model accuracy until the optimal set is determined. In this study, the following methods were employed for these evaluations.

**Logistic Regression (LR):** Forward selection, backward elimination, or recursive feature elimination (RFE) techniques can be employed. Forward selection adds features incrementally based on performance improvement, while backward elimination removes features sequentially to minimize performance reduction. RFE iteratively eliminates the least important features until the desired set is obtained.

**Random Forests (RF):** An ensemble learning method that aggregates predictions from multiple decision trees. Feature importance scores can be obtained by measuring the average decrease in impurity across trees. Recursive Feature Elimination (RFE) can be applied using these scores to iteratively remove less important features.

##### Embedded Approaches

Embedded methods integrate feature selection directly into the model training process, determining the most significant features during model construction. Algorithms used include Linear Discriminant Analysis (LDA), Bagged Decision Trees, and LASSO, which inherently incorporate feature selection as part of their learning algorithms.

**LDA:** Linear Discriminant Analysis (LDA) maximizes the ratio of between-class variance to within-class variance to determine the best features. It is formulated as follows:

(9) $$J\left(W\right)=\frac{\left|W^{T}S_{b}W\right|}{\left|W^{T}S_{w}W\right|},$$

where $S_{b}$ represents the between-class scatter matrix, $S_{w}$ is the within-class scatter matrix, and *W* is the projection matrix.

**LASSO:** Least Absolute Shrinkage and Selection Operator (LASSO) regression performs feature selection by adding an L1 regularization term to the following loss function:

(10) $$\min_{\beta }\left[\sum_{i=1}^{n}{\left(y_{i}-\beta_{0}-\sum_{j=1}^{p}\beta_{j}\;x_{ij}\right)}^{2}+\lambda \sum_{j=1}^{p}\left|\beta_{j}\right|\right],$$

where λ represents the regularization parameter and $\beta_{j}$ are the regression coefficients.

This ensures that only the most relevant features are selected, which, in turn, enhances the overall performance of the models built.

#### 2.3.4. Modeling

##### Model Development

**Model Selection:** Using the right combination of features and optimum modeling becomes a crucial part of a predictive modeling research. This study compared the performance of seven different machine learning models under various feature set combinations, namely:

- Logistic Regression (LR);
- Random Forest Classifier (RF);
- XGBoost Classifier;
- Decision Tree Classifier (DT);
- K-Nearest Neighbor (KNN);
- Support Vector Machines (SVM);
- Naive Bayes [20] (NB).

These include using the 30 highest ranking continuous features plus the 20 highest ranking categorical features as determined by feature selection, and assessing how the model performs across feature sets ranging from 1 to 50 features.

For the four selected meta-learning models (LR, RF, XGBoost, and DT), Recursive Feature Elimination (RFE) was implemented—a feature selection technique that recursively trains the model and removes the least informative feature at each iteration until an optimal number (including removal of all features) is found.

However, for the remaining models, an exhaustive assessment of all possible feature combinations was conducted, given their importance to the model.

To ensure the robustness of the results, 5-fold cross-validation was performed for each model [21]. These metrics are defined as follows:

(11) $$\begin{array}{cc} \mathrm{Accuracy} & =\frac{TP+TN}{TP+TN+FP+FN}, \end{array}$$

(12) $$\begin{array}{cc} \mathrm{Precision} & =\frac{TP}{TP+FP}, \end{array}$$

(13) $$\begin{array}{cc} \mathrm{Recall}/\mathrm{Sensibility} & =\frac{TP}{TP+FN}, \end{array}$$

(14) $$\begin{array}{cc} F1-\mathrm{Score} & =2\times \frac{\mathrm{Precision}\times \mathrm{Recall}}{\mathrm{Precision}+\mathrm{Recall}}, \end{array}$$

where TP represents True Positives, TN True Negatives, FP False Positives, and FN False Negatives, respectively.

**Figure 2:**
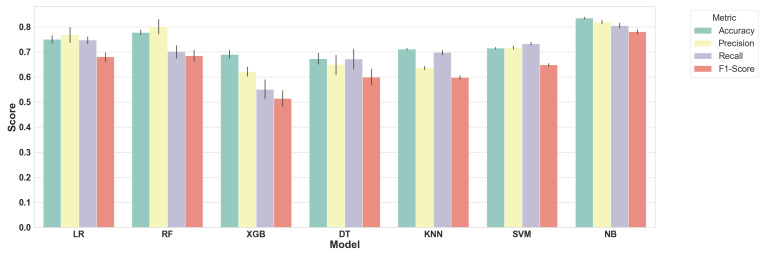
Data analysis and model development workflow diagram.

As illustrated in Figure 2, the distribution of the various parameter values for all of the models is displayed. The NB model consistently outperformed other models across all evaluated metrics, indicating its superior overall performance. SVM also demonstrated robust results, closely following NB. In contrast, models like XGBoost and RF exhibited relatively lower performance, particularly in Precision and F1-Score. As a result, further development and tuning focused on the NB model.

**Classification Model:** Upon selecting the NB model, it was refined by optimizing both the number of features and the hyperparameters. The approach systematically explored various combinations of discrete, binary, and continuous features, evaluating up to a total of 50 features to determine the optimal feature mix for improving model performance.

The final model employed a stacking approach, integrating predictions from three separate NB classifiers: GaussianNB for continuous features, MultinomialNB for discrete features, and BernoulliNB for binary features. Each classifier was independently trained on its respective feature subset.

To enhance the overall accuracy and robustness, various meta-classifiers were tested, including LR, SVC with different kernels, and other ensemble methods. The meta-classifier was trained on the combined predictions from the individual NB classifiers. This stacking approach allowed the meta-classifier to learn and correct the weaknesses of the base classifiers, thereby improving the final prediction. The meta-classifier effectively leverages the strengths of each base classifier, ensuring a more robust and accurate overall model.

##### Cross-Validation

To ensure the optimal configuration of the model, Leave-One-Out Cross-Validation (LOOCV) was employed, a rigorous validation method particularly useful for small datasets [22]. LOOCV provides a comprehensive evaluation by using each sample as a test set once, ensuring that the model is thoroughly tested.

The model’s performance was evaluated using key metrics, including Accuracy, Precision, Recall, and F1-Score, providing a detailed assessment of each feature combination. In each iteration, the individual classifiers (GaussianNB, MultinomialNB, and BernoulliNB) were trained, and their predictions were combined. The meta-classifier was then trained on these combined predictions, and performance metrics were computed for each fold and averaged to ensure robustness.

Given the clinical setting of prostate cancer management, where the cost of false positives is extremely high, prioritizing precision as the critical outcome is essential. This focus ensures that treatments are recommended only for patients most likely to benefit, enhancing both the precision and safety of clinical decisions.

The optimization process involved grid search methods to systematically vary model parameters and identify the optimal configuration. The final model achieved a balance between sensitivity and specificity, tailored to prioritize precision in clinical recommendations.

## 3. Results

### 3.1. Statistical Analysis

This analysis revealed significant patterns within both imaging and biological data that were crucial for guiding the feature selection process. Statistical analyses demonstrated distinct differences in Choline uptake in the liver and bone, which were strongly correlated with patient responses. These parameters emerged as significant predictors, indicating a substantial impact on treatment efficacy. In particular, the differences between PSMA and FDG fixations revealed a statistically significant relationship, suggesting that these differences are crucial potential factors affecting patient outcomes. Additionally, the difference between PSMA and Choline uptake showed a *p*-value of 0.0505, which is very close to the significance threshold. This near-significant result strengthens the conclusion that tracer behavior is an important factor to consider in predictive models.

In addition, several biological parameters including leukocytes, neutrophils, and alkaline phosphatase (ALP) were identified as significant factors. These findings emphasize the potential high performance of combining imaging and biological factors in predictive models. Table 3 lists the variables for which the *p*-value was less than 0.05, indicating a statistically significant difference or association with the binary class label (FBTP/NFBTP).

### 3.2. Model Development and Evaluation

The initial feature selection process identified the most informative features from the dataset. The final model optimization phase selected 14 key features, blending discrete, binary, and continuous variables. These selected features were critical in constructing the predictive model, encompassing both imaging and biological parameters.

The imaging parameters included Std. dev: g/mL_Choline_Bone+, Min: g/mL_Choline_ Liver, Std. dev: g/mL_Choline_Bone-, Peak: g/mL_Choline_Kidney, and Peak: g/mL_ Choline_Bone-. The invasion metrics included the number of lymph node involvements in supradiaphragmatic and subdiaphragmatic regions, as well as liver involvement. Additionally, tracer discordance metrics, specifically PSMA-/FDG+ and PSMA−/choline+, were included. Biological parameters such as neutrophils, leukocytes, and alkaline phosphatase (ALP) levels were also integral to the feature set.

LOOCV ensures a comprehensive evaluation by using each sample as a test set once. Various meta-classifiers were tested, and ultimately, an SVC with a sigmoid kernel was employed. Key performance metrics, including Accuracy, Precision, Recall, and F1-Score, were calculated for each iteration and averaged to ensure robustness. The final model achieved the following metrics: Accuracy: 0.92, Precision: 0.96, Recall: 0.96, and F1-Score: 0.92.

These results highlight the model’s robustness and reliability, ensuring high precision in clinical recommendations for prostate cancer treatment.

## 4. Discussion

### 4.1. Advancements in Predictive Factors and Imaging Techniques for RLT Response in Prostate Cancer

Several studies have sought to identify predictive factors for response to RLT, examining a range of demographic, histological, biochemical, and imaging parameters. However, the evaluated factors were diverse, and the results were often divergent [9]. Using advanced machine learning techniques, this study integrated diverse data sources, including imaging and biological parameters, to build a robust predictive model. The stacking approach, combining Bayesian classifiers and Support Vector Classifiers (SVC), effectively handled the complexities of small datasets while maintaining a high accuracy and precision.

The findings of this study have significant implications for clinical decision-making in patients with metastatic castration-resistant prostate cancer (mCRPC). By identifying key factors predictive of response to 177Lu-PSMA therapy, clinicians can more accurately select the most suitable treatment strategies, thereby improving therapeutic outcomes and minimizing unnecessary side effects. For instance, a high renal uptake of Choline and specific biomarkers (such as leukocyte and neutrophil levels) were associated with poor treatment response, suggesting that this information can help clinicians to assess patient suitability before treatment and optimize therapeutic planning. Additionally, the high predictive accuracy of the machine learning model (accuracy 0.92, precision 0.96) supports the approach of personalized medicine, ensuring optimal resource utilization and better patient prognosis.

Even though few patients underwent an FDG PET scan, the findings are consistent with previous research that identifies PSMA-FDG mismatch as a negative predictive factor for RLT response, which was an exclusion criterion in the TheraP study [6]. Previous studies have developed nomograms to predict the presence of PSMA-negative but FDG-positive lesions [23]. Groener et al. showed that baseline SUVmax, SUVmean, and tumor-to-liver ratio values were associated with lesion response [24]. However, it should be noted that the intensity of PSMA uptake has been identified as a predictive factor for response in some studies [25,26]. In this study, locoregional involvement on PSMA PET data was a stronger predictive factor than the lesion uptake intensity.

Regarding prostate cancer, some authors have investigated the value of both PSMA and Choline PET for predicting treatment response, but not specifically for the response to RLT [10]. Choline PET, despite lower accuracy than PSMA PET, remains a valuable diagnostic tool, particularly for advanced PCa [27]. According to Laudicella et al. [27] [18F]Choline PET/CT can improve patient selection for RLT by highlighting PSMA-non avid lesions, which aligns with the presented study as the difference between PSMA and Choline uptake approached significance (p=0.0505).

Integrating imaging data, particularly Choline PET, can significantly enhance the selection of patients for radioligand therapy (RLT). Choline PET provides detailed information on tumor metabolism and distribution, complementing PSMA PET, and helps to identify PSMA-negative but Choline-positive lesions, allowing the exclusion of patients unlikely to respond favorably to RLT. This study found that high uptake in renal and bone regions on Choline PET was correlated with poorer treatment outcomes, indicating that Choline PET is a valuable screening tool. The inclusion of Choline PET data aids in a comprehensive evaluation of tumor heterogeneity, improving the accuracy of predictive models, optimizing treatment strategies, and enhancing both survival and quality of life for patients.

To date, no study has specifically incorporated [18F]Choline PET data to predict RLT response. In the present study, [18F]Choline PET/CT features, particularly related to background renal region uptake intensity, were among the most significant predictors. The feature engineering and model optimization process ensured that the most relevant variables were selected, leading to a model with an impressive accuracy of 0.92. This highlights the importance of leveraging machine learning techniques to enhance predictive accuracy in clinical settings. The background renal uptake was notably higher in the NFBTP group compared to the FBTP group, with no apparent explanation, especially considering the absence of significant differences in the biological assessment of renal function. The intensity of uptake in the most bone Choline-avid lesions was significantly higher in NFBTP compared to FBTP, which may correlate with the aggressiveness of the tumor. From a biological perspective, elevated levels of leukocytes and neutrophils in the bloodstream are indicative of a potential systemic inflammatory response. The alkaline phosphatase level is higher in NFBTP than FBTP; the alkaline phosphatase level is known as an important biomarker for the aggressiveness of the disease and the presence of bone metastases.

Gafita et al. [28] developed a nomogram to predict outcomes after [177Lu]Lu-PSMA treatment in patients with mCRPC, including factors such as time since initial diagnosis, chemotherapy status, baseline hemoglobin, and [68Ga]Ga-PSMA-11 PET/CT parameters. This model has been validated and can aid in determining whether Lu-PSMA therapy or cabazitaxel is more likely to achieve a serum PSA response [29].

### 4.2. Study Limitations and Potential Insights from [18F]Choline PET/CT

In this study, software for quantifying bone tumor volume invasion was not available; instead, volumes were measured visually. Another limitation of this study is the small size of the cohorts, which may affect the generalizability of the findings. However, the significant features identified, especially from [18F]Choline PET/CT, underscore the potential of these parameters in predicting RLT response.

## 5. Conclusions

This study highlights the significant potential of integrating imaging and biological data to predict patient responses to radioligand therapy (RLT) with [177Lu]Lu-PSMA in metastatic castration-resistant prostate cancer (mCRPC) patients. Utilizing a stacking model that combines Bayesian classifiers and SVC enhanced the predictive accuracy and reliability of the model. The findings underscore the importance of using comprehensive datasets, including [18F]Choline PET, [68Ga]Ga-PSMA-11 PET, and [18F]FDG PET scans, along with biological parameters such as neutrophils, leukocytes, and alkaline phosphatase levels.

The rigorous data analysis and feature selection process, guided by Leave-One-Out Cross-Validation (LOOCV), ensured that the most relevant features were selected for model development. The final model achieved an impressive accuracy of 0.92, a precision of 0.96, a recall of 0.96, and an F1-score of 0.92, underscoring the robustness and clinical utility of the proposed approach.

The results emphasize the critical role of Choline PET in providing unique insights into the predictive factors affecting RLT efficacy. Additionally, the identified statistical differences in Choline uptake and tracer discordance (PSMA−/FDG+) serve as potential indicators for patient stratification. These findings can significantly contribute to refining patient selection processes and optimizing treatment planning.

To validate the predictive factors identified in this study, future research should involve larger sample sizes and multicenter, prospective studies to enhance the generalizability and statistical significance of the results. Additionally, longer follow-up periods are necessary to assess the association of predictive factors with long-term survival, progression-free survival (PFS), and overall survival (OS), further confirming their clinical utility. Moreover, combining other imaging modalities (such as MRI) with Choline PET should be explored to understand the synergistic role of multimodal imaging in predicting treatment response, thus enhancing the predictive power of the model.

In conclusion, this study not only enhances the understanding of the predictive factors influencing RLT effectiveness but also demonstrates the powerful application of machine learning techniques in personalized cancer treatment. The proposed framework can be broadly applied to similar small-sample, multi-feature classification tasks, paving the way for improved patient outcomes in various clinical settings.

## Figures and Tables

**Table 1:** Clinical characteristics of the patients included in the study.

Patient	Age	WHO ^a^	Gleason Score	PSA Level before RLT (ng/mL)	PSA Level after RLT (ng/mL)	Number of Cures
1	73	2	9	560	>1200	1
2	61	1	7	385	28.1	6
3	64	0	8	68	915.1	3
4	50	1	8	68.4	1636	4
5	78	0	7	14.2	1.52	6
6	83	1	6	46.79	5.88	6
7	79	1	6	47	52	4
8	75	1	10	22.4	376	5
10	85	1	7	92.77	15.86	2
11	68	1	7	37	13.87	6
12	73	1	7	0.8	0.12	5
15	75	1	8	198.4	152	2
16	77	1	7	87.31	7	6
19	79	1	7	51.7	158.1	5
20	63	1	9	21.99	3.8	6
21	83	1	8	65.263	0.4	6
22	73	2	8	27.11	<0.006	6
23	80	2	8	98.42	3.82	6
24	75	1	9	134.59	243.82	4
28	60	1	9	172.68	243	1
30	64	1	7	19.3	35.42	6
31	74	0	7	77.28	2	6
32	70	0	NA ^b^	42.55	1.49	6
33	78	1	8	93.3	179	6
36	63	1	9	2009	>5000	3

^a^ World Health Organization performance status; ^b^ no Gleason score (diagnostic on biopsy of lymph node).

**Table 2:** Descriptions of the parameters.

Parameters		Description
Radiomics Features	Imaging Parameters	Statistical features such as mean, minimum, maximum, peak values of ROI
	Invasion	Bone, pelvic tissue, liver, lung, and lymph node invasion
	Tracer Status	Consistency and fixed states of PSMA, Choline, and FDG tracers
Clinical Parameters	Age	Patient’s age (range from 50 to 85)
	Gleason Score	Describes abnormality degree of cancer cells in prostate (range from 6 to 10)
	TNM Staging	Tumor size, lymph node involvement, and metastasis staging
	WHO Classification	World Health Organization classification of the disease
Biological Parameters	PSA Level	Baseline serum prostate-specific antigen level
	Complete Blood Count	Hemoglobin, Leukocytes, Neutrophils, Lymphocytes, Platelets
	Liver Function Tests	ASAT, ALAT, Total Bilirubin, Albumin, ALP
	Kidney Function Tests	GFR, Creatinine

ROI, regions of interest; ASAT, aspartate aminotransferase; ALAT, alanine aminotransferase; ALP, alkaline phosphatase; GFR, glomerular filtration rate.

**Table 3:** Significant variables and corresponding *p*-values.

Variable	FBTP	NFBTP ^a^	*p*-Value
Max: g/mL_Choline_Kidney	14.97 (3.33)	18.34 (3.25)	0.013
Min: g/mL_Choline_Kidney	6.30 (1.41)	7.72 (1.37)	0.013
Mean: g/mL_Choline_Kidney	9.64 (2.27)	11.91 (2.35)	0.023
Peak: g/mL_Choline_Kidney	11.79 (2.54)	14.84 (2.82)	0.013
Std. dev: g/mL_Choline_Kidney	1.85 (0.50)	2.36 (0.51)	0.021
Max: g/mL_Choline_Bone+ ^b^	6.91 (6.57)	11.81 (5.98)	0.040
Min: g/mL_Choline_Bone+	2.92 (2.79)	4.98 (2.53)	0.035
Mean: g/mL_Choline_Bone+	4.17 (4.29)	7.36 (3.92)	0.035
Peak: g/mL_Choline_Bone+	4.86 (4.75)	7.94 (3.52)	0.035
Std. dev: g/mL_Choline_Bone+	0.98 (1.08)	1.71 (1.03)	0.027
Leukocytes (G/L)	5.23 [4.65, 5.96]	6.66 [5.60, 8.43]	0.013
Neutrophils (G/L)	3.29 [2.77, 3.64]	4.05 [3.13, 5.79]	0.040
ALP (Alkaline Phosphatase)	91.00 [62.00, 112.00]	188.50 [95.50, 351.50]	0.035
Invasion score of Pelvis ^c^	1.00 [0.00, 3.00]	5.50 [3.25, 7.88]	0.033
Difference_PSMA-FDG ^d^	(0.5/0/0.5)	(0/0.67/0.33)	0.025
Difference_PSMA-choline	(0.64/0.27/0.09)	(0.5/0.29/0.21)	0.0505 ^e^

^a^ Values are presented as mean (SD) for normally distributed continuous variables, median [IQR] for non-normally distributed continuous variables, and frequencies of (no difference, less, greater) for categorical variables. ^b^ Bone+ refers to bone regions with higher radiotracer uptake. ^c^ The invasion score of the pelvis is a measure of the extent of cancer invasion in the pelvic region. ^d^ Discordance between PSMA and FDG was categorized as no difference, PSMA less than FDG, and PSMA more than FDG. These were recorded as difference_PSMA-FDG to analyze their relationship with treatment response. ^e^ The *p*-value approaches the significance threshold but does not meet it.

## Data Availability

The datasets used in this study are available from the corresponding authors upon reasonable request.

## Abbreviations

The following abbreviations are used in this manuscript:

PC	Prostate cancer
PSMA	Prostate specific membrane antigen
FDG	Fluorodeoxyglucose
RLT	Radioligand therapy
ML	Machine learning
